# Macrophages—Target and Tool in Tumor Treatment: Insights from Ovarian Cancer

**DOI:** 10.3390/cancers17193182

**Published:** 2025-09-30

**Authors:** Małgorzata Górczak, Łukasz Kiraga

**Affiliations:** 1 Center of Cellular Immunotherapies, Warsaw University of Life Sciences, 02-787 Warsaw, Poland; malgorzata_gorczak@sggw.edu.pl; 2 Division of Pharmacology and Toxicology, Department of Preclinical Sciences, Institute of Veterinary Medicine, Warsaw University of Life Sciences, 02-787 Warsaw, Poland

**Keywords:** ovarian cancer, macrophages, immunotherapy, CAR-M, CAR-T

## Abstract

**Simple Summary:**

Ovarian cancer is a highly aggressive malignancy with significant treatment challenges, especially given the limited success of current treatments. Our investigation highlights that macrophages, which are abundant within tumors, frequently drive cancer progression and resistance to therapy, but they can also be reprogrammed to exert anti-tumor effects or utilized as anti-cancer agents in novel immunotherapies. In this review, we focus on the dual therapeutic potential of macrophages—as both a target and a tool. As a target, strategies include inhibiting their recruitment, selectively depleting tumor-associated macrophages (TAMs), or reprogramming them toward tumoricidal phenotypes. As a tool, macrophages are being engineered into Chimeric Antigen Receptor Macrophages (CAR-Ms) or employed as delivery vehicles for anti-cancer agents. A particularly promising innovation is the development of Macrophage–Drug Conjugates (MDCs), which exploit the transfer of iron-binding proteins (TRAIN) mechanism for precise intracellular drug delivery. These macrophage-based approaches hold significant promise for advancing more effective treatments for ovarian cancer and other solid tumors.

**Abstract:**

Today, science and medicine are striving to develop novel techniques for treating deadly diseases, including a wide range of cancers. Efforts are being made to better understand the molecular and biochemical mechanisms of tumor cell functioning, but a particular emphasis has recently been given to investigating immune cells residing in the tumor microenvironment, which may lead to revolutionary benefits in the design of new immunotherapies. Among these cells, tumor-associated macrophages (TAMs) are highly abundant and act as critical regulators of ovarian cancer progression, metastasis, and resistance to therapy. Their dual nature—as drivers of malignancy and as potential therapeutic mediators—has positioned them at the forefront of research into next-generation immunotherapies. As therapeutic targets, approaches include blocking macrophage recruitment (e.g., CSF-1/CSF-1R inhibitors), selectively depleting subsets of TAMs (e.g., via Folate Receptor Beta), or reprogramming immunosuppressive M2-like macrophages toward an anti-tumor M1 phenotype. On the other hand, macrophages can also serve as a therapeutic tool—they may be engineered to enhance anti-tumor immunity, as exemplified by the development of Chimeric Antigen Receptor Macrophages (CAR-Ms), or leveraged as delivery vehicles for targeted drug transport into the tumor microenvironment. A particularly innovative strategy involves Macrophage–Drug Conjugates (MDCs), which employs the transfer of iron-binding proteins (TRAIN) mechanism for precise intracellular delivery of therapeutic agents, thereby enhancing drug efficacy while minimizing systemic toxicity. This review integrates current knowledge of TAM biology, highlights emerging therapeutic approaches, and underscores the promise of macrophage-based interventions in ovarian cancer. By integrating macrophage-targeting strategies with advanced immunotherapeutic platforms, novel treatment paradigms may be determined that could substantially improve outcomes for patients with ovarian cancer and other solid tumors. Our work highlights that macrophages should be a particular area of research interest in the context of cancer treatment.

## 1. Introduction—Clinical Significance of Macrophages in Ovarian Cancer

Ovarian cancer is the leading cause of death associated with gynecologic cancers, largely due to its late diagnosis and limited biomarkers for early detection. Despite advances in surgery and chemotherapy, recurrence is common, and treatment options are still limited. Novel strategies such as CD40 agonists, dendritic cell vaccines and T cell receptor (TCR) engineering are currently being investigated to enhance anti-tumor immune responses. However, recent studies highlight the importance of the tumor microenvironment (TME) in driving tumor progression, therapy resistance and metastasis. In the TME, immune cells, particularly macrophages, have been identified as critical regulators of ovarian cancer malignancy [1].

Macrophages are a heterogeneous population of immune cells that play an important role in both innate immunity and tissue homeostasis. They exhibit intrinsic plasticity and can adopt various states of activation depending on environmental factors. Macrophages display remarkable plasticity, ranging from the M1 state, linked to inflammatory and anti-cancer effects, to the M2 state, which is associated with immune suppression and supports tumor development [2]. In TME of ovarian cancer, the predominant presence of macrophages with M2 polarity is often observed, where they contribute to tumor progression, metastasis and tumor resistance to therapies [3,4].

M2 macrophages present in the microenvironment of ovarian cancer are known to facilitate tumor progression through multiple mechanisms. They promote angiogenesis by secreting vascular endothelial growth factor (VEGF) and other pro-angiogenic agents, supporting the development of a vascular system that promotes tumor growth [5]. Moreover, M2 macrophages facilitate tumor cell invasion and metastasis by secreting matrix metalloproteinases (MMPs) that degrade the proteins of extracellular matrix [6]. In addition to their pro-proliferative effects, M2 macrophages also provide an immunosuppressive microenvironment by secreting cytokines such as IL-10 and TGF-β, which contribute to the suppression of anti-tumor immunity and promote tumor cell immune evasion [7].

Given the significant biological role of macrophages in ovarian cancer progression, targeting macrophage polarization or their recruitment to the tumor site has emerged as a promising strategy for therapeutic approaches. Recent studies have explored the potential of reprogramming M2 macrophages into their more tumor-suppressive M1 phenotype as a novel immunotherapeutic approach [8,9]. Additionally, blocking macrophage recruitment with inhibitors of chemokine signaling pathways has shown promise in preclinical models of ovarian cancer [10]. Thus, macrophages are known to play a significant role in shaping the TME of ovarian cancer, and their polarization affects tumor progression, metastasis and therapeutic resistance. These findings underscore the potential of macrophages as both prognostic biomarkers and novel targets for ovarian cancer therapy. Targeting macrophage phenotype represents a promising route to enhance current treatment strategies which might improve ovarian cancer outcomes.

## 2. The Biology of Ovarian Cancer

### 2.1. Epidemiology and Pathogenesis of Ovarian Cancer

Epithelial ovarian cancer (EOC) is a prevalent form of ovarian cancer, with High-Grade Serous Carcinoma (HGSC) being the most lethal tumor of the female genital tract and a significant subtype [11,12]. The incidence of ovarian cancer appears higher in developed countries, which may be partly due to better detection and reporting, as well as differences in lifestyle and reproductive factors [13,14].

The risk of ovarian cancer increases significantly in individuals with inherited mutations, primarily in the *BRCA1* and *BRCA2* genes [12,15,16]. These genes encode breast cancer type 1 and 2 susceptibility proteins [15]. For carriers of *BRCA1* or *BRCA2* pathogenic variants, the lifetime risk for ovarian cancer can be 39–44% and 11–17%, respectively [15,17]. It is noted that germline BRCA mutations are found in approximately 3.5% of ovarian cancer patients, and 10–20% of ovarian cancer cases may be associated with them [18,19]. Risk is also elevated by mutations in other homologous recombination repair (HRR) pathway genes, including *BRIP1*, *PALB2*, *RAD51C*, and *RAD51D* [20,21,22]. Furthermore, mutations in the *TP53* gene are extremely common in HGSC, occurring in approximately 96% of cases [23]. These mutations lead to the loss of *TP53*’s suppressor function, contributing to genomic instability and tumor progression [24]

The latest data on ovarian cancer incidence and mortality in different regions show significant discrepancies, which can be linked to differences in access to appropriate treatment (Table 1) [12].

Recent epidemiological studies highlight disparities in incidence and survival in different populations, emphasizing the role of genetic and environmental factor, among others [13]. Major risk factors contributing to ovarian cancer pathogenesis are listed in Table 2.

Advances in genetic screening and early detection programs are helping to mitigate mortality rates in high-risk populations. Early detection biomarkers, such as circulating tumor DNA (ctDNA), exosomal RNA, and microRNA panels, are being explored to improve diagnosis at earlier stages with a more favorable prognosis [26]. Efforts to implement population-wide genetic screening and risk assessment strategies are gaining popularity, especially in high-risk groups. In addition, ongoing studies aim to elucidate the impact of the microbiome, immune dysregulation and epigenetic modifications on the development of ovarian cancer [12].

### 2.2. Molecular Characterization and Development

Molecular profiling of ovarian cancer has identified distinct subtypes based on genetic, transcriptomic, and epigenetic landscapes. HGSC is characterized by homologous recombination deficiency (HRD) characterized by *TP53* mutation and frequent alterations in genes such as *BRCA1*, *BRCA2*, and *RAD51C*. Low-grade serous carcinoma (LGSC) exhibits mutations in *KRAS*, *BRAF*, and *NRAS* genes, demonstrating a different molecular trajectory. Epigenetic modifications, including DNA methylation and histone acetylation, play a key role in regulating gene expression and are being explored as potential therapeutic targets [27,28,29].

Single-cell RNA sequencing (scRNA-seq), proteomic analyses and spatial transcriptomics have improved our understanding of tumor heterogeneity, revealing distinct cellular subpopulations contributing to chemoresistance and metastasis [30,31].

### 2.3. Prognostic Factors

Prognosis in ovarian cancer is largely dependent on tumor stage, molecular profile, and treatment response. Key prognostic markers impacting survival are given in Table 3.

### 2.4. Composition of the Tumor Microenvironment (TME) and the Immune Cells Compartment in Ovarian Cancer

The tumor microenvironment (TME) is a highly complex and dynamic system that plays a critical role in tumor initiation, progression, immune evasion, and therapeutic resistance [32]. In solid tumors, alongside malignant cells, the TME includes a heterogeneous population of non-malignant cells, such as endothelial cells, adipocytes, cancer-associated fibroblasts (CAFs), tumor associated macrophages (TAMs), infiltrating monocytes, and granulocytes, as well as structural components like the extracellular matrix (ECM) [33,34]. This environment is shaped by the local secretion of chemokines (e.g., CCL2, CXCL12) and growth factors (e.g., CSF-1, VEGF), which coordinate the recruitment, polarization, and functional reprogramming of these cells in ways that ultimately promote tumor progression [35].

**Figure 1:**
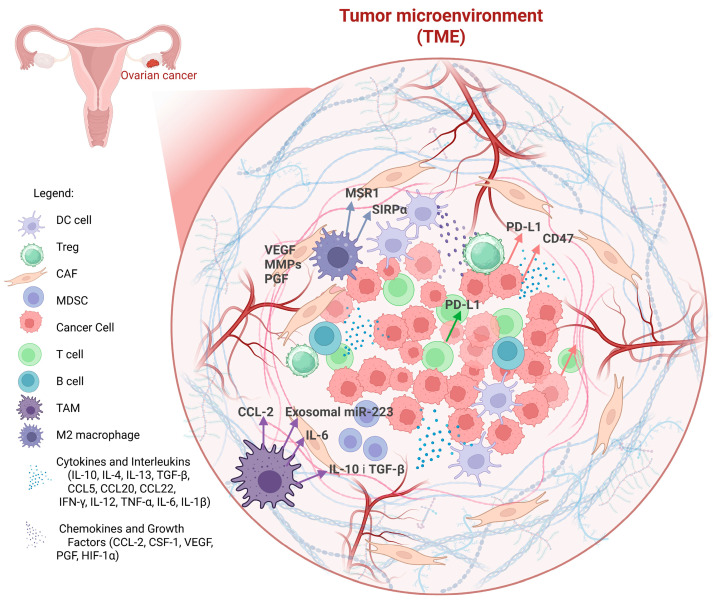
The Ovarian Cancer Tumor Microenvironment (TME) and its Key Components.

A schematic of the microenvironment of ovarian cancer including the immune cell niche is shown in Figure 1. The TME is a complex ecosystem surrounding the tumor, playing a critical role in tumor progression and response to therapy.

Schematic illustration of the ovarian cancer TME, highlighting the complex cellular and molecular interactions that support tumor progression and immune evasion. The TME contains multiple immune and stromal components, including T cells, B cells, dendritic cells (DCs), regulatory T cells (Tregs), myeloid-derived suppressor cells (MDSCs), tumor-associated macrophages (TAMs), and cancer-associated fibroblasts (CAFs). A key feature is the predominance of M2-like TAMs, which secrete immunosuppressive cytokines (IL-10, TGF-β), chemokines (CCL-2, CCL20, CCL22), and growth factors (VEGF, PGF, CSF-1, HIF-1α), fostering angiogenesis, tumor growth, and suppression of cytotoxic immunity. Immune checkpoint molecules, including PD-L1 expressed on tumor cells and TAMs, and CD47–SIRPα signaling, further inhibit T cell and macrophage activity, while exosomal miR-223 and IL-6 contribute to tumor-promoting inflammation. Collectively, these mechanisms create a highly immunosuppressive microenvironment that hinders anti-tumor immune responses and supports ovarian cancer progression.

Tumor-associated macrophages (TAMs), regulatory T cells (Tregs) and myeloid-derived suppressor cells (MDSCs) contribute to immune evasion and poor prognosis. Recent studies utilizing spatial transcriptomics have identified immune “hot” and “cold” tumor microenvironments, which affect immunotherapy differently (Table 4) [36,37].

The immune landscape is a crucial component of the tumor microenvironment. In ovarian cancer, this landscape is heterogeneous, with individual cellular subpopulations influencing disease progression and therapeutic response. In particular, TAMs play a major role in it.

### 2.5. Characteristics and Biological Functions of Macrophages

Macrophages represent a heterogeneous population of mononuclear immune cells that differ in their localization, phenotype, morphology, and gene expression profiles [38]. For decades, it was assumed that macrophages arise exclusively through the differentiation of circulating monocytes in peripheral blood. However, subsequent studies revealed significant morphological and functional differences between these cell types, challenging this long-standing view. It is now recognized that the majority of mature tissue-resident macrophages originate during embryogenesis, deriving from yolk sac progenitors, and possess the capacity for self-renewal [34]. Additional evidence for their developmental independence comes from the observation that macrophage levels remain stable in tissues of patients with monocytopenia, despite the reduction in circulating monocytes. Thus, most macrophages in healthy tissues are of prenatal origin and persist independently of the hematopoietic system [39].

Nonetheless, macrophages can also arise from monocytes in adulthood, particularly under pathological conditions such as inflammation. Monocytes originate from the bone marrow, developing from hematopoietic stem cells, and account for approximately 4–10% of peripheral blood mononuclear cells. They have a relatively short half-life of about 20 h. During inflammatory responses, monocytes exit the circulation, migrate into tissues, and differentiate into macrophages. In this context, they serve as a reservoir for replenishing tissue-resident phagocytes. Unlike embryonically derived macrophages, monocyte-derived macrophages are generally short-lived and lack self-renewal capacity [38,39].

A key feature of both monocytes and macrophages is their ability to migrate to sites of inflammation or injury, where they play a critical role in eliminating the cause of tissue damage and initiating repair processes [40]. Recruitment is driven by cytokines and chemokines secreted by activated T cells, with monocyte chemoattractant protein-1 (MCP-1) being a central regulator [41]. MCP-1, released by fibroblasts, endothelial cells, and T lymphocytes, induces firm adhesion of monocytes to the vascular endothelium and facilitates their transmigration into damaged tissues, representing a fundamental immune defense mechanism [38].

Another hallmark of macrophages is their remarkable plasticity, which enables them to adopt distinct phenotypes in response to signals from the tissue microenvironment. Cytokines secreted by Th1 or Th2 lymphocytes drive polarization into classically activated (M1) or alternatively activated (M2) macrophage subsets [38,39,41,42]. This process is tightly regulated by transcription factors that determine functional specialization. M1 polarization is mediated by STAT-1 in response to interferon-γ (IFN-γ), by STAT-1/STAT-2 heterodimers induced by bacterial lipopolysaccharide (LPS), and by nuclear factor kB (NF-kB). In contrast, M2 polarization involves STAT-3 and STAT-6, activated by cytokines such as IL-4, IL-13, and IL-10 [42].

### 2.6. Origin and Pathophysiological Role of TAMs

Monocytes migrating from the bloodstream into the tumor site differentiate under the influence of local signals into TAMs [34]. In the early stages of tumor development, macrophages often exhibit a classically activated, M1-like phenotype, characterized by the production of pro-inflammatory and anti-tumor cytokines such as IFN-γ and IL-12 [43]. However, as the tumor evolves, persistent exposure to tumor-derived signals shifts macrophage polarization toward an alternatively activated, M2-like phenotype. M2-like TAMs represent a dominant immune cell population in the TME and play multifaceted roles in promoting tumor growth. They secrete large amounts of CCL2, which perpetuates the recruitment of additional monocytes, thereby maintaining and amplifying the TAM population. The abundance of CCL2 correlates positively with TAM density, tumor stage, and poor clinical prognosis [44,45,46].

In addition to TAM density and polarization, several novel biomarkers are being identified to better stratify ovarian cancer patients for TAM-targeted therapies, enabling a more refined and personalized approach to treatment selection. Macrophage-specific targets include HER2 overexpression, which has shown to predict a more favorable response to HER2-directed CAR-macrophage (CT-0508) therapy, with stable disease observed exclusively in HER2 IHC 3+ tumors [47]. Similarly, the presence of Folate Receptor Beta (FRβ) on TAMs is a key indicator for FRβ-targeted selective macrophage depletion strategies, such as those employing monoclonal antibodies like m909 [48,49]. Other macrophage-associated checkpoints, such as CD47/SIRPα, are also under investigation, with CD47 inhibitors (e.g., magrolimab) aiming to enhance macrophage phagocytic activity and bispecific molecules like SL-172154 combining CD47 blockade with CD40 activation [50,51,52]. Strategies like CSF-1 receptor (CSF1R) inhibition are being evaluated to reprogram immunosuppressive TAMs, making CSF1R a potential stratification marker for such interventions [53]. Furthermore, the tumor microenvironment offers broader immune context biomarkers, including immunosuppressive metabolic enzymes like Arginase 1 (ARG1) and CD73, the latter generating adenosine that inhibits T-cells, prompting the development of CD73 or A2A adenosine receptor antagonists [54]. Chemokine and cytokine profiles, alongside transcriptomic immune signatures, such as an “inflamed” gene signature characterized by high expression of CXCL9, CD8A, and interferon-gamma (IFN-γ)–related genes, are being explored as correlates of immune responsiveness. Lastly, spatial features like the presence of tertiary lymphoid structures (TLS) within tumors have been associated with improved immune responses and could offer complementary tools for patient stratification. The integration of these diverse molecular and spatial biomarkers is crucial for advancing personalized macrophage-centric treatment approaches in ovarian cancer, addressing the current challenge of limited predictive biomarkers for immunotherapy [55,56].

TAMs contribute significantly to the immunosuppressive landscape of the TME. They impair cytotoxic T lymphocyte (CTL) function through the expression of inhibitory surface molecules, secretion of immunosuppressive chemokines (e.g., CCL5, CCL20, CCL22), and generation of reactive oxygen species (ROS) [57,58]. These mechanisms not only inhibit effective anti-tumor immunity but also promote the recruitment of regulatory T cells (Tregs), further dampening the immune response [59]. In addition to their immunomodulatory functions, TAMs enhance tumor angiogenesis and metastatic potential by producing pro-angiogenic factors such as vascular endothelial growth factor (VEGF) and placental growth factor (PGF), as well as matrix-degrading enzymes like matrix metalloproteinases (MMP-2, MMP-7, MMP-9). These molecules remodel the ECM and facilitate neovascularization, thereby creating conditions conducive to tumor cell invasion and dissemination [60].

In the following sections, we will cover specific signaling pathways involved in the interactions between TAMs and ovarian cancer cells, and discuss emerging therapeutic interventions based on targeting TAMs in ovarian cancer.

#### Interaction Between TAMs and Ovarian Tumor Cells—Recruitment and Characteristics

TAMs play a key role in ovarian cancer progression by promoting tumor invasion, promoting tumor angiogenesis and contributing to treatment resistance. TAMs are mainly derived from circulating monocytes and residual macrophages that are recruited to the tumor microenvironment (TME) in response to various chemotactic signals released by ovarian tumor cells. The recruitment of monocytes to the ovarian tumor site is primarily mediated by tumor-derived cytokines and chemokines such as:

- Colony-stimulating factor-1 (CSF-1)—drives the differentiation of monocytes into macrophages [61].
- C-C motif chemokine ligand 2 (CCL2)—enhances monocyte recruitment to the TME [62].
- Vascular endothelial growth factor (VEGF) and hypoxia-inducible factor-1α (HIF-1α)—create a hypoxic and angiogenic environment that attracts and polarizes macrophages towards a pro-tumor phenotype [63].

After reaching the TME, monocytes differentiate into TAMs under the influence of tumor-derived factors such as IL-10, TGF-β and lactic acid. TAMs in ovarian cancer often exhibit an M2-like phenotype, which is associated with immune suppression, tissue remodeling and tumor progression. TAMs in the ovarian cancer microenvironment exhibit several pro-tumorigenic properties as shown in Table 5.

The interaction between ovarian cancer cells and TAMs is a dynamic and reciprocal process that facilitates tumor progression. Tumor cells release signals that recruit and polarize macrophages, while TAMs, in turn, provide survival advantages to the tumor by modulating the immune system, enhancing invasion, and stimulating angiogenesis. This complex interplay is one of the key reasons behind treatment failure in ovarian cancer.

From a clinical perspective, targeting TAMs offers a promising strategy to improve treatment efficacy. Ideally, interventions would aim to reduce the number or suppress the function of immunosuppressive M2-like TAMs, while enhancing the activity or prevalence of pro-inflammatory M1-like macrophages [68]. Several therapeutic approaches are currently under investigation, including blocking the migration and recruitment of macrophages into the tumor microenvironment, depleting macrophages, reprogramming TAMs toward M1-like phenotype, disrupting TAM-cancer cell signaling pathways as well as inhibiting immune checkpoints. [69,70].

While TAM-targeted therapies are most likely to be effective as adjuvants, enhancing the activity of checkpoint inhibitors, chemotherapy, or PARP inhibitors, there is also emerging evidence that they may exert stand-alone effects in certain settings [44]. For instance, blockade of the CD47/SIRPα axis can promote direct tumor cell phagocytosis by macrophages, while CSF1R inhibition can reduce the abundance of immunosuppressive TAMs and reprogram them toward a pro-inflammatory phenotype [71]. However, given the highly redundant immunosuppressive networks in the ovarian tumor microenvironment, durable clinical benefit is more likely when TAM-targeted strategies are combined with checkpoint blockade or integrated into current standards of care such as surgery, chemotherapy, and PARP inhibitors. Thus, TAM-directed therapy may serve a stand-alone role in select contexts, but its greatest potential lies in synergistic, combination therapies [72].

Understanding these interactions provides insight into potential therapeutic strategies of TAMs targeting. By disrupting these cancer-promoting mechanisms, we may potentially improve outcomes for ovarian cancer patients.

## 3. Macrophages—Target in Ovarian Cancer Therapy

### 3.1. Blocking the Migration and Recruitment of Macrophages into the Tumor Microenvironment

Various strategies of targeting tumor-associated macrophages (TAMs) have been investigated in recent clinical trials to enhance anti-cancer therapy. One approach involves blocking the migration and recruitment of macrophages into the tumor microenvironment. One of the most explored strategies involves blocking the colony-stimulating factor 1 (CSF-1) or its receptor (CSF-1R), which are essential for the differentiation and survival of macrophages. Inhibition of the CSF-1/CSF-1R axis can both reduce monocyte-to-macrophage differentiation and promote the depletion of established TAMs, thereby reshaping the immunological landscape of the tumor in favor of anti-tumor immunity [73,74]. Therapeutic blockade of CSF-1/CSF-1R signaling has been shown to limit the survival of tumor-supportive macrophages and to strengthen anti-tumor immune activity [75].

In another study, targeting the CCL2-CCR2 axis has shown promise in reducing TAM infiltration, thereby limiting tumor progression [76].

### 3.2. Reprogramming TAMs

Another strategy focuses on reprogramming TAM from a pro-cancer M2 phenotype to an anti-cancer M1 phenotype. Agents targeting the CD47-SIRPα interaction, such as Hu5F9-G4, aim to promote macrophage-mediated phagocytosis of tumor cells through conversion to M1. Clinical trials have shown that blocking CD47 is a promising strategy for cancer treatment [77].

### 3.3. Selective Depletion of Macrophages

Selective depletion of macrophages, particularly TAMs, has emerged as a promising strategy to enhance anti-cancer therapy in ovarian cancer (OC). TAMs are highly abundant in the OC tumor microenvironment (TME) and contribute significantly to immune suppression and tumor progression. One key approach involves targeting folate receptor beta (FRβ) on TAMs [49]. Monoclonal antibodies, such as m909, have shown preclinical efficacy in targeting FRβ-expressing TAMs to selectively deplete these immunosuppressive macrophages and promote a more immune-permissive environment. By reducing the number or suppressing the function of immunosuppressive M2-like TAMs, while enhancing the activity or prevalence of pro-inflammatory M1-like macrophages, these interventions aim to improve treatment efficacy [78].

### 3.4. Blocking Immune Checkpoints

Current therapies target immune checkpoint inhibitors (ICIs) such as cytotoxic T-lymphocyte antigen 4 (CTLA-4) and programmed cell death receptor 1 (PD-1) and its ligand PD-L1 [79,80]. The first FDA-approved checkpoint blockade therapy was the human monoclonal antibody anti-CTLA-4, ipilimumab, used in melanoma treatment [81]. CTLA-4 acts as a coinhibitory receptor that inhibits T cell activation. Subsequent checkpoint-targeting agents include anti-PD-1 antibodies (pembrolizumab and nivolumab), which demonstrate a better safety profile than anti-CTLA-4. PD-1 and its ligand PD-L1 are coinhibitory molecules that regulate T cell responses at the tumor cell surface. Tumor cells expressing PD-L1 bind to PD-1 receptors on T cells, leading to inhibition of their cytotoxic activity against tumor cells. The use of anti-PD-1 or anti-PD-L1 antibodies prevents this immune suppression, thereby sustaining T cell-mediated anti-tumor responses [80,82].

Recent studies indicate that TAMs can also express PD-1, particularly under conditions of chronic immune stimulation and predominantly within the immunosuppressive M2-like subset [83]. PD-1 signaling in TAMs can suppress their phagocytic activity and the production of pro-inflammatory cytokines, further contributing to tumor immune evasion. Consequently, PD-1/PD-L1 blockade may have dual effects: restoring T cell cytotoxicity and reprogramming TAMs towards a more pro-inflammatory, anti-tumor phenotype [84]. Blocking immune checkpoints like PD-1/PD-L1 has also been explored to enhance macrophage-mediated anti-tumor responses. Immune checkpoint inhibitors (ICIs) have shown promise, but their efficacy remains limited due to the immunosuppressive effects of the TME. However, combining PD-1/PD-L1 inhibitors with other TAM-targeting therapies has shown synergistic effects in preclinical models, suggesting potential for improved clinical outcomes [83]. The previously discussed advancements underscore the potential of TAM-targeted therapies in improving clinical outcomes for cancer patients.

### 3.5. Effectiveness of TAM-Targeted Therapy—Summary

Therapies targeting tumor-associated macrophages (TAMs) are increasingly being investigated across various solid tumors. To frame in the ovarian cancer context, we provide a comparative overview of selected solid tumors, highlighting therapeutic strategies and observed outcomes (Table 6).

The ovarian tumor microenvironment is characterized by high TAM infiltration, which significantly supports tumor growth and therapy resistance [97]. Preliminary data indicate that TAM-targeted interventions in ovarian cancer may exhibit greater efficacy than in other solid tumors, such as glioblastoma, pancreatic, or lung cancer, where immunosuppressive environments or dense stroma limit therapeutic outcomes [98].

Breast cancer also demonstrates promising responsiveness to TAM-targeted therapies, particularly when combined with chemotherapy or immunotherapy, due to the role of TAMs in promoting metastasis and immune evasion [99]. Challenges common across all solid tumors include macrophage heterogeneity, adaptive recruitment of immunosuppressive cells, and interactions with other components of the tumor microenvironment. As a result, TAM-targeted strategies often achieve maximal efficacy in combination with other treatments rather than as monotherapy.

## 4. Macrophages—A Tool in Ovarian Cancer Therapy

On the other hand, macrophages may also play a role in the fight against ovarian cancer as a component of cellular immunotherapies.

In light of current research, the use of chimeric antigen receptor macrophages (CAR-M) and macrophages as delivery vehicles seems particularly promising.

### 4.1. Chimeric Antigen Receptor (CAR)

Several CAR generations exist, differing mainly in their intracellular signaling domains. First-generation CARs contain only the CD3ζ signaling domain but show limited activity as T cell activation also requires costimulatory signals from CD3 and CD28 complexes. Second-generation CARs incorporate an additional costimulatory domain such as CD28 or 4-1BB, while third-generation CARs include two costimulatory domains (CD28 and 4-1BB) [100,101]. Fourth-generation CARs, known as T cells redirected for universal cytokine killing (TRUCKs), enhance anti-tumor efficacy by releasing cytokines, antibodies (e.g., anti-PD-1), and enzymes capable of degrading the extracellular matrix in solid tumors [102]. For example, TRUCKs release IL-12 upon antigen recognition, which stimulates T cells and increases IFN-γ secretion within the tumor microenvironment [102,103].

While most CAR research has focused on T and NK cells, CAR-expressing macrophages (CAR-M) are emerging as a promising approach for solid tumors. CAR-Ms combine inherent tissue infiltration and phagocytic activity with the ability to modulate the tumor microenvironment and present tumor antigens to T cells, bridging innate and adaptive immunity [104]. Recent preclinical and early clinical studies in ovarian cancer have shown that CAR-Ms targeting mesothelin, HER2, or CD47 can phagocytose tumor cells, inhibit tumor growth, and enhance T cell and NK cell infiltration [105]. These findings suggest that CAR-M therapy could overcome some limitations of CAR-T approaches in solid tumors. Ongoing research aims to optimize CAR design, delivery methods, and the ability of CAR-Ms to remodel immunosuppressive tumor microenvironments, highlighting their potential as a next-generation immunotherapy for ovarian cancer.

### 4.2. CAR-M

Engineered macrophages equipped with CARs (CAR-M) are gaining attention as they can penetrate solid tumors more effectively than T cells and maintain anti-cancer activity within the tumor microenvironment [106]. Tumor-associated macrophages (TAMs) frequently outnumber T cell infiltrates in tumors and are recruited by chemokines (CCL2, CXCL12), colony-stimulating factor 1 (CSF-1), and VEGF [107]. This natural abundance and tumor-homing ability give CAR-M therapies an advantage in penetrating solid tumors where CAR-T cells struggle.

Studies have shown that CAR macrophages can efficiently phagocytose and present tumor antigens, modulate the immunosuppressive tumor microenvironment, and stimulate adaptive immunity. For instance, Klichinsky et al. (2020) demonstrated that HER2-targeted CAR macrophages could infiltrate solid tumors, induce pro-inflammatory responses, and suppress tumor growth in preclinical models [108]. Building on this, interim results from a phase I clinical trial (NCT04660929) evaluating autologous HER2-targeted CAR macrophages (CT-0508) in patients with advanced HER2-expressing solid tumors have recently been published [62]. The study reported a favorable safety profile with no dose-limiting toxicities, ≥grade 3 cytokine release syndrome, or immune effector cell-associated neurotoxicity syndrome. Among patients with HER2 IHC 3+ tumors, 44% achieved stable disease at 8 weeks, while no clinical activity was observed in the IHC 2+ cohort. Biopsies confirmed tumor infiltration by CT-0508 and associated increases in intratumoral CD8+ T cell infiltration, supporting the translational potential of CAR macrophages in the treatment of solid tumors [109].

CAR-M may have superior efficacy compared to CAR-T in solid tumors due to their ability to remodel the tumor microenvironment and overcome immunosuppressive barriers. Their phagocytic activity and persistence suggest they could complement or even outperform traditional CAR-T therapies in certain cancers, especially those with complex tumor stroma. Challenges for CAR-M include overcoming the tumor’s immunosuppressive signals that polarize macrophages toward a tumor-promoting M2 phenotype, and the need to identify highly specific tumor antigens to avoid off-target effects. Current research is focused on engineering CAR macrophages to sustain anti-tumor immunity by reprogramming the tumor microenvironment [110,111].

Overall, CAR-based therapies face obstacles related to antigen specificity and the complexity of the tumor microenvironment, especially in epithelial-origin solid tumors where distinguishing malignant from healthy cells is critical for safety and efficacy [112].

### 4.3. Macrophages as a Delivery Vehicle

Macrophages have garnered considerable attention as potential vehicles for therapeutic delivery due to their innate ability to home to sites of inflammation, infection, and tumors. This natural tropism toward pathological environments makes macrophages uniquely suited for targeted drug delivery, especially in solid tumors, where they can constitute up to 50% of the tumor mass. Their phagocytic capacity allows them to uptake significantly larger quantities of therapeutic compounds compared to other cell types [113].

One innovative strategy involves engineering macrophages to carry nanoparticles loaded with drugs or genetic material. These cells possess the ability to traverse complex biological environments and cross physiological barriers, such as the blood–brain barrier, to deliver therapeutic cargo directly to the tumor site. Drug internalization can occur via endocytosis or phagocytosis, while subsequent release may proceed through passive diffusion or active transport mechanisms.

Macrophages can also be genetically or chemically modified to express surface receptors or ligands, enhancing their specificity for certain tissues or tumor cell populations. This targeted delivery capability is particularly beneficial in oncological applications, where precise localization of chemotherapeutic agents is essential to maximize efficacy while minimizing systemic toxicity [114].

Several preclinical studies have demonstrated the feasibility of using macrophages in this context. For instance, Muthana and colleagues developed a system utilizing M1-polarized macrophages (autologous) to deliver a hypoxia-activated oncolytic adenovirus for the treatment of prostate cancer, resulting in significant tumor growth inhibition [115,116,117]. In another study, macrophages were used to transport liposomal doxorubicin in a non-small cell lung cancer model (A549), where five administrations of macrophage-carried liposomal doxorubicin produced a more pronounced tumor growth suppression than either free drug or liposomes delivered via conventional methods [118].

Beyond the use of modified cells, unmodified macrophages have also been investigated for drug delivery. A recent breakthrough in macrophage-based therapies is the development of Macrophage–Drug Conjugates (MDCs), as described by Taciak et al. (2025) [119]. This platform uses allogenic macrophages loaded with chemotherapeutic agents encapsulated within human heavy-chain ferritin (HFt) nanocages. The HFt-drug complex is efficiently internalized by macrophages via MSR1-mediated endocytosis, and subsequently transferred to cancer cells through a unique, contact-dependent mechanism termed TRAIN (TRAnsfer of Iron-binding proteiN). TRAIN relies on the formation of an immune synapse-like interface between macrophages and tumor cells, enabling direct intracellular delivery of the drug payload. Preclinical studies across multiple tumor models—including ovarian, lung, and pancreatic cancers—have demonstrated that MDCs reduce tumor growth, improve survival, and retain therapeutic efficacy even after cryopreservation, supporting their potential as an off-the-shelf allogeneic cell therapy for solid tumors [119,120].

In a recent study focusing on glioblastoma, one of the most challenging and aggressive primary brain tumors, an innovative adoptive cell therapy based on macrophage-ferritin-drug conjugates, specifically named MDC-735, has shown promising activity. In complex glioblastoma patient samples obtained directly from surgery, macrophages demonstrated a preferential transfer of ferritin-conjugates to cancerous cells over non-malignant cells from the tumor microenvironment. Additionally, MDC-735 induced morphological changes in glioma cells and showed marked phagocytic activity against glioma cells, suggesting enhanced effector function [121].

Encapsulation of cytotoxic drugs directly within macrophages is generally unfeasible due to the high toxicity of these compounds to the carrier cells. However, the use of ferritin as a biocompatible carrier protects both the macrophage and the drug, preventing lysosomal degradation within the host cell. This is a unique property of macrophages, as ferritin is rapidly degraded in other cell types [122]. The MDC technology aims to enhance the precision and efficacy of chemotherapy, reduce systemic toxicity, and improve drug bioavailability in otherwise resistant tumor niches [119,121].

**Figure 2:**
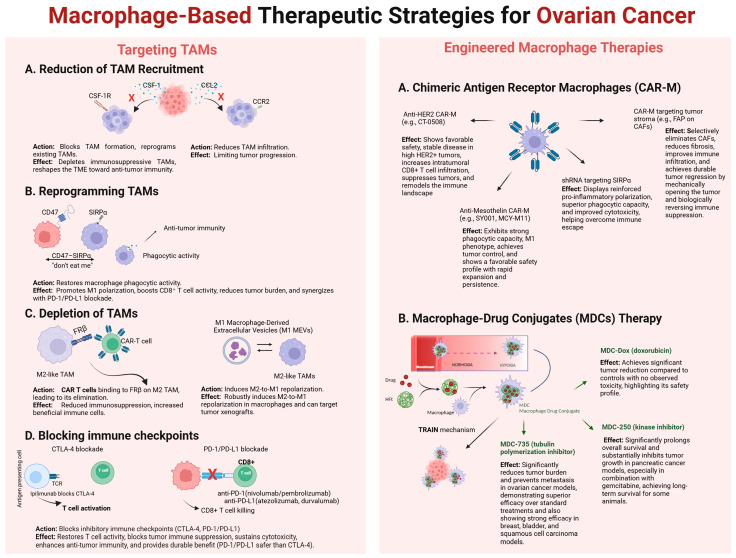
A summary of ovarian cancer treatment methods utilizing macrophages as a therapeutic target or therapeutic agent.

The growing understanding of ovarian cancer biology, enabled by advances in immunological and molecular profiling, supports the development of personalized immunotherapeutic strategies. In this context, the use of macrophages as active components of drug delivery systems represents a promising and safe frontier in targeted cancer therapy. On the other hand, macrophages can also serve as a target in immunotherapy for various solid tumors, including ovarian cancer. A summary of ovarian cancer treatment methods utilizing macrophages as a therapeutic target or therapeutic agent is shown schematically in Figure 2.

Schematic representation of current macrophage-based strategies aimed at overcoming the immunosuppressive tumor microenvironment (TME) in ovarian cancer. I. Targeting TAMs. Approaches include: (A) Reduction in TAM recruitment by blocking CSF-1/CSF-1R and CCL2/CCR2 signaling; (B) Reprogramming TAMs via agents such as M1 macrophage-derived extracellular vesicles (M1 MEVs) to shift M2-like macrophages toward an anti-tumor M1 phenotype; (C) Depletion of TAMs using CAR T cells directed against FRβ on M2-like TAMs; (D) Immune checkpoint blockade targeting CTLA-4, PD-1/PD-L1, and CD47–SIRPα pathways to restore T cell and macrophage activity. II. Engineered Macrophage Therapies. Advanced therapeutic include: (A) Chimeric Antigen Receptor Macrophages (CAR-Ms), such as anti-HER2 CAR-M (CT-0508), anti-mesothelin CAR-M (SY001, MCY-M11), and CAR-M targeting tumor stroma (e.g., FAP on CAFs), which promote phagocytosis, enhance CD8+ T cell infiltration, reduce fibrosis, and remodel the immune landscape; (B) Macrophage–Drug Conjugates (MDCs), where macrophages deliver therapeutic payloads (e.g., MDC-735, MDC-250, MDC-Dox) through the TRAIN mechanism, enabling drug release under normoxic and hypoxic conditions, significantly reducing tumor burden and preventing metastasis with minimal toxicity.

### 4.4. CAR-M and MDC Therapies: Divergent Challenges and Emerging Opportunities

Translating CAR-macrophage (CAR-M) and macrophage–drug conjugate (MDC) therapies into clinical practice faces substantial scientific, logistical, and regulatory challenges. For CAR-Ms, a persistent limitation is the immunosuppressive tumor microenvironment (TME), which frequently drives infused macrophages toward an M2-like phenotype, thereby diminishing their antitumor activity and necessitating strategies to sustain pro-inflammatory M1 polarization in vivo [105,123]. In addition, CAR-Ms often exhibit limited persistence and expansion, particularly when engineered with transient mRNA platforms, leading to short-lived responses. Antigen specificity poses another hurdle: many tumor-associated antigens are broadly expressed across healthy tissues, raising the risk of on-target/off-tumor toxicity. To mitigate this, approaches such as dual-antigen recognition, logic-gated CARs, and cytokine co-engineering are under investigation. Manufacturing also remains complex—autologous CAR-Ms are patient-specific, expensive, and time-intensive, while viral delivery methods raise safety concerns, including insertional mutagenesis. Induced pluripotent stem cell (iPSC)-derived CAR-Ms hold promise as scalable “off-the-shelf” alternatives, but maintaining their stable phenotype and functionality within the TME remains an open challenge. Overall, CAR-Ms remain at an early stage of development, with ongoing concerns regarding safety, efficacy optimization, and clinical applicability [108]. By contrast, MDCs are supported by extensive preclinical evidence that highlights their translational readiness. Mechanistically, they exploit the TRAIN (TRAnsfer of Iron-binding proteiN) pathway, in which macrophages internalize drug-loaded heavy-chain ferritin (HFt) via clathrin-mediated endocytosis and the scavenger receptor MSR1, then transfer HFt directly to tumor cells through a synapse-like interface resembling virological synapses. This unique biology enables highly selective intercellular delivery of cytotoxic payloads, while minimizing extracellular diffusion and off-target effects. Preclinical studies have validated the consistency of this approach across diverse macrophage sources, including human primary macrophages, iPSC-derived macrophages, and established cell lines.

The therapeutic potential of MDCs has been demonstrated in multiple solid tumor models. MDC-735, for example, inhibited tumor growth and metastasis in ovarian cancer (SK-OV-3) and breast cancer models, while MDC-250 significantly prolonged survival in pancreatic cancer, also with gemcitabine in combination therapy. Moreover, allogeneic MDC-735 combined with anti-PD-1 therapy achieved notable tumor regression and survival benefit in bladder (MB49) and squamous cell carcinoma (SCC7) models, supporting its compatibility with checkpoint blockade. Importantly, MDCs deliver potent anticancer activity even at lower drug doses where free drug showed no efficacy, highlighting their ability to enhance therapeutic index through selective tumor targeting [119].

According to our unpublished research safety and capacity for large-scale production further set apart MDCs from other cell therapies. Macrophages retained viability and function after drug loading and long-term cryopreservation, reinforcing the feasibility of MDCs as true “off-the-shelf” products. Their favorable safety profile was confirmed even at high cell doses, and selective cytotoxicity against cancer cells while preserving healthy tissues was consistently observed in co-culture systems. Preclinical evaluation showed no signs of graft-versus-host disease (GvHD) or systemic toxicity following allogeneic administration, with comprehensive histopathological analyses of major organs revealing no abnormalities [119]. Together, these findings demonstrate that MDCs not only overcome several critical hurdles associated with CAR-Ms, such as antigen specificity, persistence, and manufacturing complexity, but also introduce a unique drug delivery paradigm rooted in macrophage physiology.

## 5. Conclusions

Macrophages appear to be particularly interesting in the context of the treatment of solid tumors, including an especially malignant one—ovarian cancer. Based on current knowledge of the biology of macrophages residing in the tumor microenvironment, we know that macrophages can be considered a potential target in cancer therapy. By triggering the mechanisms described earlier, this approach can lead to the suppression of various types of tumors and, with high probability, might exhibit the highest efficacy in ovarian cancer. On the other hand, macrophages can also be tools in cellular immunotherapies, especially when they are given to the patient via adoptive therapy as CAR-M. TAM-targeting strategies can function both as an adjuvant to existing immunotherapies, such as checkpoint inhibitors—enhancing T cell infiltration, reducing immunosuppressive signals, and potentiating anti-tumor responses—and as a potential standalone approach in technologies like MDC involving the TRAIN technique, which aim to directly reprogram macrophages toward a pro-inflammatory, anti-tumor phenotype.

However, the MDC technology involving the TRAIN technique seems particularly promising in the context of treating ovarian cancer and other types of tumors. Nevertheless, conclusive data from clinical trials are needed to confirm the high efficacy of the described approaches.

## Figures and Tables

**Table 1:** Regional Cancer Incidence and Mortality Rates (per 100,000 Population).

Region	Incidence	Mortality Rate (Per 100,000)
North America	8.4	4.9
Europe	7.8	5.1
Asia	6.2	4.0
Africa	3.5	2.7
Latin America	5.4	3.6

**Table 2:** Several risk factors contribute to ovarian cancer pathogenesis [25].

Risk Factors	Description	Impact or Risk
Genetic Mutations	homologous recombination repair (HRR) pathway genes (*BRCA1*, *BRCA2*, *TP53*, *RAD51C*, *PALB2*)	High
Reproductive and Hormonal Factors	Early menarche, late menopause, nulliparity, hormone replacement therapy (HRT)	Moderate
Lifestyle and Environmental Factors	Smoking, obesity, high-fat diets	Low to moderate
Inflammation	Endometriosis, pelvic inflammatory disease	Moderate

**Table 3:** Key prognostic markers include.

Prognostic Factor	Impact on Survival
Tumor Microenvironment	High CD8+ T-cell infiltration correlates with better prognosis
BRCA Mutations	Improved response to PARP inhibitors and platinum-based chemotherapy
Circulating Biomarkers	ctDNA and exosomal RNA predict treatment response
Chemoresistance Genes	ABC transporters and drug efflux genes contribute to resistance
Inflammatory Markers	IL-6 and TNF-alpha associated with poor prognosis

**Table 4:** The table shows a summary of the characteristics of immune “hot” and immune “cold” tumors as identified through spatial transcriptomics [36].

Feature	Immune “Hot” Tumors	Immune “Cold” Tumors
Immune Cell Infiltration	High (especially CD8+ T cells)	Low
Gene Expression Profile	High expression of immune activation and IFN-γ pathways	Low immune gene expression; suppressive markers may dominate
Response to Immunotherapy	Often responsive	Generally unresponsive
Presence of TLS (Tertiary Lymphoid Structures)	Common	Rare or absent
Tumor Microenvironment (TME)	Inflamed, immunologically active	Immune-excluded or immunosuppressed
Common Immune Cell Types	CD8+ T cells, Th1 cells, dendritic cells	Tregs, M2 macrophages, few effector T cells
Spatial Pattern	Dense clusters of immune cells within tumor core and periphery	Sparse immune presence, often restricted to stromal edges
Therapeutic Strategy	Checkpoint inhibitors, adoptive T cell therapy	Combination therapies to induce immune infiltration

**Table 5:** Tumor-promoting activities of TAMs: functions, mechanisms, and key mediators.

Function	Mechanism	Key Factors
Promotion of Tumor Invasion	TAMs secrete matrix metalloproteinases (MMPs), particularly MMP-2 and MMP-9, which degrade the extracellular matrix (ECM) and facilitate tumor cell invasion. TAMs also enhance epithelial-to-mesenchymal transition (EMT), supporting metastasis [64,65].	MMP-2, MMP-9, EMT [64,65].
Fostering Tumor Angiogenesis	TAMs promote neovascularization by releasing VEGF, PDGF, and angiopoietins. They also secrete pro-inflammatory cytokines like TNF-α and IL-6, which stimulate endothelial proliferation and increase vascular permeability [66].	VEGF, PDGF, angiopoietins, TNF-α, IL-6 [66].
Immune Suppression and Therapy Resistance	TAMs produce immunosuppressive cytokines (IL-10, TGF-β) that inhibit cytotoxic T cells and expand regulatory T cells. They upregulate immune checkpoint molecules (e.g., PD-L1), contributing to immune evasion. TAMs also support chemotherapy resistance via interactions with cancer stem cells, aiding tumor survival and dormancy [64,67].	IL-10, TGF-β, PD-L1, cancer stem cell signaling [64,67].

**Table 6:** Therapeutic strategies and the observed outcomes of solid tumor treatment based on the specific TAM-targeted approaches.

Tumor Type	TAM-Targeted Approach	Key Observations/Effects
Glioblastoma	CSF-1R inhibitors, TAM reprogramming [85]	Monotherapy often shows limited efficacy; improved outcomes observed in combination with chemotherapy or anti-angiogenic therapy [85,86,87].
Pancreatic Cancer	TAM recruitment blockade (CCR2), CSF-1R inhibitors [88]	Moderate efficacy; outcome dependent on combination with chemotherapy [89,90].
Lung Cancer	TAM polarization reprogramming, CSF-1R inhibitors [91].	Variable results depending on histological subtype; some studies suggest synergy with immunotherapy [92].
Breast Cancer	CSF-1R inhibitors, TAM depletion or repolarization [93]	Promising preclinical results; combination with chemotherapy or immune checkpoint blockade enhances response [94].
Ovarian Cancer	CSF-1R inhibitors, TAM polarization modulation [95]	Preclinical and early clinical data suggest comparable or slightly higher efficacy relative to other solid tumors [96].

## Abbreviations

The following abbreviations are used in this manuscript:

ACT	Adoptive Cell Transfer
ADU-S100	(MIW815)—A STING agonist
BCMA	B-cell Maturation Antigen
*BRCA1*	Breast cancer type 1 susceptibility protein gene
*BRCA2*	Breast cancer type 2 susceptibility protein gene
CAFs	Cancer-Associated Fibroblasts
CAR	Chimeric Antigen Receptor
CAR-M	Chimeric Antigen Receptor Macrophages
CAR-NK	Chimeric Antigen Receptor Natural Killer cells
CAR-T	Chimeric Antigen Receptor T cells
CCL2	C-C Motif Chemokine Ligand 2
CCR2	C-C Chemokine Receptor Type 2
CSF-1	Colony-Stimulating Factor 1
CSF-1R	Colony-Stimulating Factor 1 Receptor
CTL	Cytotoxic T Lymphocytes
CTLA-4	Cytotoxic T-Lymphocyte-Associated Protein 4
DNAM-1	DNAX Accessory Molecule-1
ECM	Extracellular Matrix
EMT	Epithelial-to-Mesenchymal Transition
EOC	Epithelial Ovarian Cancer
FAP	Fibroblast Activation Protein (a CAR-M target)
FDA	Food and Drug Administration
FRβ	Folate Receptor Beta
GvHD	Graft-versus-Host Disease
HFt	Human Heavy-Chain Ferritin
HER2	Human Epidermal growth factor Receptor 2
HGSC	High-Grade Serous Carcinoma
HIF-1α	Hypoxia-Inducible Factor 1-alpha
HRR	Homologous Recombination Repair
ICIs	Immune Checkpoint Inhibitors
IFN-γ	Interferon Gamma
IL-10	Interleukin 10
iPSC	Induced Pluripotent Stem Cell
LGSC	Low-Grade Serous Carcinoma
M1	Classically activated macrophages (pro-inflammatory)
M1 MEVs	M1 macrophage-derived extracellular vesicles
M2	Alternatively activated macrophages (immunosuppressive)
MDCs	Macrophage–Drug Conjugates
MDSCs	Myeloid-Derived Suppressor Cells
MHC	Major Histocompatibility Complex
MICA/MICB	MHC Class I Chain-Related Protein A/B
MMPs	Matrix Metalloproteinases
NF-kB	Nuclear factor kB
NK	Natural Killer
PARP	Poly(ADP-ribose) Polymerase
PD-1	Programmed Cell Death Protein 1
PD-L1	Programmed Death-Ligand 1
PGF	Placental Growth Factor
RMI	Risk of Malignancy Index
ROS	Reactive Oxygen Species
TAMs	Tumor-Associated Macrophages
TCR	T Cell Receptor
TGF-β	Transforming Growth Factor Beta
TME	Tumor Microenvironment
TNF-α	Tumor Necrosis Factor Alpha
TRAIN	TRAnsfer of Iron-binding proteiN
TRUCKs	T cells redirected for universal cytokine-mediated killing
Tregs	Regulatory T Cells
ULBP	UL16-Binding Proteins
VEGF	Vascular Endothelial Growth Factor
ctDNA	Circulating Tumor DNA
scFv	Single-Chain Variable Fragment
scRNA-seq	Single-Cell RNA Sequencing

## References

[1] Mantovani A., Sica A. (2010). Macrophages, innate immunity and cancer: Balance, tolerance, and diversity. Curr. Opin. Immunol..

[2] Sica A., Mantovani A. (2012). Macrophage plasticity and polarization: In vivo veritas. J. Clin. Investig..

[3] Carey P., Low E., Harper E., Stack M.S. (2021). Metalloproteinases in Ovarian Cancer. Int. J. Mol. Sci..

[4] Zhang Q., Li H., Mao Y., Wang X., Zhang X., Yu X., Tian J., Lei Z., Li C., Han Q. (2019). Apoptotic SKOV3 cells stimulate M0 macrophages to differentiate into M2 macrophages and promote the proliferation and migration of ovarian cancer cells by activating the ERK signaling pathway. Int. J. Mol. Med..

[5] Bertout J.A., Patel S.A., Simon M.C. (2008). The impact of O_2_ availability on human cancer. Nat. Rev. Cancer.

[6] Niland S., Riscanevo A.X., Eble J.A. (2021). Matrix Metalloproteinases Shape the Tumor Microenvironment in Cancer Progression. Int. J. Mol. Sci..

[7] Yang Y., Yang Y., Yang J., Zhao X., Wei X. (2020). Tumor Microenvironment in Ovarian Cancer: Function and Therapeutic Strategy. Front. Cell Dev. Biol..

[8] Xu C., Chen J., Tan M., Tan Q. (2025). The role of macrophage polarization in ovarian cancer: From molecular mechanism to therapeutic potentials. Front. Immunol..

[9] Cao W., Chen H.-D., Yu Y.-W., Li N., Chen W.-Q. (2021). Changing profiles of cancer burden worldwide and in China: A secondary analysis of the global cancer statistics 2020. Chin. Med, J..

[10] Reddy J.P., Atkinson R.L., Larson R., Burks J.K., Smith D., Debeb B.G., Ruffell B., Creighton C.J., Bambhroliya A., Reuben J.M. (2018). Mammary stem cell and macrophage markers are enriched in normal tissue adjacent to inflammatory breast cancer. Breast Cancer Res. Treat..

[11] Desai A., Xu J., Aysola K., Qin Y., Okoli C., Hariprasad R., Chinemerem U., Gates C., Reddy A., Danner O. (2014). Epithelial ovarian cancer: An overview. World J. Transl. Med..

[12] Wilczyński J., Paradowska E., Wilczyński M. (2024). High-Grade Serous Ovarian Cancer—A Risk Factor Puzzle and Screening Fugitive. Biomedicines.

[13] Momenimovahed Z., Tiznobaik A., Taheri S., Salehiniya H. (2019). Ovarian cancer in the world: Epidemiology and risk factors. Int. J. Women’s Health.

[14] Gaona-Luviano P., Medina-Gaona L.A., Magaña-Pérez K. (2020). Epidemiology of ovarian cancer. Chin. Clin. Oncol..

[15] Petrucelli N., Daly M.B., Pal T. (1998). BRCA1- and BRCA2-Associated Hereditary Breast and Ovarian Cancer.

[16] Huang J., Chen J., Huang Q. (2018). Diagnostic value of HE4 in ovarian cancer: A meta-analysis. Eur. J. Obstet. Gynecol. Reprod. Biol..

[17] Antoniou A., Pharoah P.D., Narod S., Risch H.A., Eyfjord J.E., Hopper J.L., Loman N., Olsson H., Johannsson O., Borg A. (2003). Average Risks of Breast and Ovarian Cancer Associated with *BRCA1* or *BRCA2* Mutations Detected in Case Series Unselected for Family History: A Combined Analysis of 22 Studies. Am. J. Hum. Genet..

[18] Iqbal J., Ragone A., Lubinski J., Lynch H.T., Moller P., Ghadirian P., Foulkes W.D., Armel S., Eisen A., Neuhausen S.L. (2012). The incidence of pancreatic cancer in *BRCA1* and *BRCA2* mutation carriers. Br. J. Cancer.

[19] Jeong K.-Y., Park M.H. (2021). The Significance of Targeting Poly (ADP-Ribose) Polymerase-1 in Pancreatic Cancer for Providing a New Therapeutic Paradigm. Int. J. Mol. Sci..

[20] Lisio M.-A., Fu L., Goyeneche A., Gao Z.-H., Telleria C. (2019). High-Grade Serous Ovarian Cancer: Basic Sciences, Clinical and Therapeutic Standpoints. Int. J. Mol. Sci..

[21] Song H., Dicks E., Ramus S.J., Tyrer J.P., Intermaggio M.P., Hayward J., Edlund C.K., Conti D., Harrington P., Fraser L. (2015). Contribution of Germline Mutations in the *RAD51B*, *RAD51C*, and *RAD51D* Genes to Ovarian Cancer in the Population. J. Clin. Oncol..

[22] Graffeo R., Rana H., Conforti F., Bonanni B., Cardoso M., Paluch-Shimon S., Pagani O., Goldhirsch A., Partridge A., Lambertini M. (2022). Moderate penetrance genes complicate genetic testing for breast cancer diagnosis: *ATM*, *CHEK2*, *BARD1* and *RAD51D*. Breast.

[23] Davar R., Yalamanchili M. (2022). Identification of a Panel of Biomarkers for the Early Detection of Ovarian Cancer. J. Stud. Res..

[24] Ngu S.F., Chai Y.K., Choi K.M., Leung T.W., Li J., Kwok G.S.T., Chu M.M.Y., Tse K.Y., Cheung V.Y.T., Ngan H.Y.S. (2022). Diagnostic Performance of Risk of Malignancy Algorithm (ROMA), Risk of Malignancy Index (RMI) and Expert Ultrasound Assessment in a Pelvic Mass Classified as Inconclusive by International Ovarian Tumour Analysis (IOTA) Simple Rules. Cancers.

[25] Ali A.T., Al-Ani O., Al-Ani F. (2023). Epidemiology and risk factors for ovarian cancer. Menopausal Rev..

[26] Zhang L., Hu C., Huang Z., Li Z., Zhang Q., He Y. (2021). In Silico screening of circulating tumor DNA, circulating microRNAs, and long non-coding RNAs as diagnostic molecular biomarkers in ovarian cancer: A comprehensive meta-analysis. PLoS ONE.

[27] Haunschild C.E., Tewari K.S. (2021). The current landscape of molecular profiling in the treatment of epithelial ovarian cancer. Gynecol. Oncol..

[28] Jaliffa C., Rogel U., Sen I., Singer G. (2024). Comprehensive Genomic Characterization in Ovarian Low-Grade and Chemosensitive and Chemoresistant High-Grade Serous Carcinomas. Oncology.

[29] Hollis R.L., Gourley C. (2016). Genetic and molecular changes in ovarian cancer. Cancer Biol. Med..

[30] Eriksson G., Li C., Sparovec T.G., Dekanski A., Torstensson S., Risal S., Ohlsson C., Hirschberg A.L., Petropoulos S., Deng Q. (2025). Single-cell profiling of the human endometrium in polycystic ovary syndrome. Nat. Med..

[31] Xiang X., Tao X., Hua K., Jiang H., Ding J. (2025). Single-cell RNA sequencing reveals tumor heterogeneity in small cell neuroendocrine cervical carcinoma. Commun. Biol..

[32] Avci C.B., Bagca B.G., Nikanfar M., Takanlou L.S., Takanlou M.S., Nourazarian A. (2024). Tumor microenvironment and cancer metastasis: Molecular mechanisms and therapeutic implications. Front. Pharmacol..

[33] de Visser K.E., Joyce J.A. (2023). The evolving tumor microenvironment: From cancer initiation to metastatic outgrowth. Cancer Cell.

[34] Padzińska-Pruszyńska I.B., Taciak B., Kiraga Ł., Smolarska A., Górczak M., Kucharzewska P., Kubiak M., Szeliga J., Matejuk A., Król M. (2024). Targeting Cancer: Microenvironment and Immunotherapy Innovations. Int. J. Mol. Sci..

[35] Kloosterman D.J., Akkari L. (2023). Macrophages at the interface of the co-evolving cancer ecosystem. Cell.

[36] Tai Y.-T., Lin W.-C., Ye J., Chen D.T.-H., Chen K.-C., Wang D.Y.-T., Tan T.Z., Wei L.-H., Huang R.Y.-J. (2024). Spatial Profiling of Ovarian Clear Cell Carcinoma Reveals Immune-Hot Features. Mod. Pathol..

[37] Wu B., Zhang B., Li B., Wu H., Jiang M. (2024). Cold and hot tumors: From molecular mechanisms to targeted therapy. Signal Transduct. Target. Ther..

[38] Shapouri-Moghaddam A., Mohammadian S., Vazini H., Taghadosi M., Esmaeili S.-A., Mardani F., Seifi B., Mohammadi A., Afshari J.T., Sahebkar A. (2018). Macrophage plasticity, polarization, and function in health and disease. J. Cell. Physiol..

[39] Varol C., Mildner A., Jung S. (2015). Macrophages: Development and Tissue Specialization. Annu. Rev. Immunol..

[40] Vogel D.Y., Heijnen P.D., Breur M., de Vries H.E., Tool A.T., Amor S., Dijkstra C.D. (2014). Macrophages migrate in an activation-dependent manner to chemokines involved in neuroinflammation. J. Neuroinflamm..

[41] Curi R., de Siqueira Mendes R., de Campos Crispin L.A., Norata G.D., Sampaio S.C., Newsholme P. (2017). A past and present overview of macrophage metabolism and functional outcomes. Clin. Sci..

[42] Tugal D., Liao X., Jain M.K. (2013). Transcriptional Control of Macrophage Polarization. Arterioscler. Thromb. Vasc. Biol..

[43] Rongvaux A., Willinger T., Martinek J., Strowig T., Gearty S.V., Teichmann L.L., Saito Y., Marches F., Halene S., Palucka A.K. (2014). Development and function of human innate immune cells in a humanized mouse model. Nat. Biotechnol..

[44] Tong N., He Z., Ma Y., Wang Z., Huang Z., Cao H., Xu L., Zou Y., Wang W., Yi C. (2021). Tumor Associated Macrophages, as the Dominant Immune Cells, Are an Indispensable Target for Immunologically Cold Tumor-Glioma Therapy?. Front. Cell Dev. Biol..

[45] Saeed A.F. (2025). Tumor-Associated Macrophages: Polarization, Immunoregulation, and Immunotherapy. Cells.

[46] Ghamangiz S., Jafari A., Maleki-Kakelar H., Azimi H., Mazloomi E. (2025). Reprogram to heal: Macrophage phenotypes as living therapeutics. Life Sci..

[47] Xu J., Ding L., Mei J., Hu Y., Kong X., Dai S., Bu T., Xiao Q., Ding K. (2025). Dual roles and therapeutic targeting of tumor-associated macrophages in tumor microenvironments. Signal Transduct. Target. Ther..

[48] Rodriguez-Garcia A., Lynn R.C., Poussin M., Eiva M.A., Shaw L.C., O’cOnnor R.S., Minutolo N.G., Casado-Medrano V., Lopez G., Matsuyama T. (2021). CAR-T cell-mediated depletion of immunosuppressive tumor-associated macrophages promotes endogenous antitumor immunity and augments adoptive immunotherapy. Nat. Commun..

[49] Roy A.G., Robinson J.M., Sharma P., Rodriguez-Garcia A., Poussin M.A., Nickerson-Nutter C., Powell D.J. (2021). Folate Receptor Beta as a Direct and Indirect Target for Antibody-Based Cancer Immunotherapy. Int. J. Mol. Sci..

[50] Dizman N., Buchbinder E.I. (2021). Cancer Therapy Targeting CD47/SIRPα. Cancers.

[51] Sikic B.I., Lakhani N., Patnaik A., Shah S.A., Chandana S.R., Rasco D., Colevas A.D., O’rOurke T., Narayanan S., Papadopoulos K. (2019). First-in-Human, First-in-Class Phase I Trial of the Anti-CD47 Antibody Hu5F9-G4 in Patients with Advanced Cancers. J. Clin. Oncol..

[52] Lakhani N.J., Stewart D., Richardson D.L., Dockery L.E., Van Le L., Call J., Rangwala F., Wang G., Ma B., Metenou S. (2025). First-in-human phase I trial of the bispecific CD47 inhibitor and CD40 agonist Fc-fusion protein, SL-172154 in patients with platinum-resistant ovarian cancer. J. Immunother. Cancer.

[53] Yu M., Wu Y., Li Q., Hong W., Hu X., Yang Y., Lu T., Zhao X., Wei X. (2024). Colony-stimulating factor-1 receptor inhibition combined with paclitaxel exerts effective antitumor effects in the treatment of ovarian cancer. Genes Dis..

[54] Gaudreau P.-O., Allard B., Turcotte M., Stagg J. (2016). CD73-adenosine reduces immune responses and survival in ovarian cancer patients. OncoImmunology.

[55] Alcaraz-Sanabria A., Baliu-Piqué M., Saiz-Ladera C., Rojas K., Manzano A., Marquina G., Casado A., Cimas F.J., Pérez-Segura P., Pandiella A. (2020). Genomic Signatures of Immune Activation Predict Outcome in Advanced Stages of Ovarian Cancer and Basal-Like Breast Tumors. Front. Oncol..

[56] Sun G., Liu Y. (2024). Tertiary lymphoid structures in ovarian cancer. Front. Immunol..

[57] Zeng X.-Y., Xie H., Yuan J., Jiang X.-Y., Yong J.-H., Zeng D., Dou Y.-Y., Xiao S.-S. (2019). M2-like tumor-associated macrophages-secreted EGF promotes epithelial ovarian cancer metastasis via activating EGFR-ERK signaling and suppressing lncRNA LIMT expression. Cancer Biol. Ther..

[58] Bialasek M., Kubiak M., Gorczak M., Braniewska A., Kucharzewska-Siembieda P., Krol M., Taciak B. (2019). Exploiting Iron-Binding Proteins for Drug Delivery. Ournal Physiol. Pharmacol..

[59] Kirkham P. (2007). Oxidative stress and macrophage function: A failure to resolve the inflammatory response. Biochem. Soc. Trans..

[60] Liu D., Liu L., Zhao X., Zhang X., Chen X., Che X., Wu G. (2025). A comprehensive review on targeting diverse immune cells for anticancer therapy: Beyond immune checkpoint inhibitors. Crit. Rev. Oncol..

[61] Chanmee T., Ontong P., Konno K., Itano N. (2014). Tumor-Associated Macrophages as Major Players in the Tumor Microenvironment. Cancers.

[62] DeNardo D.G., Ruffell B. (2019). Macrophages as regulators of tumour immunity and immunotherapy. Nat. Rev. Immunol..

[63] Truxova I., Cibula D., Spisek R., Fucikova J. (2023). Targeting tumor-associated macrophages for successful immunotherapy of ovarian carcinoma. J. Immunother. Cancer.

[64] Steitz A.M., Steffes A., Finkernagel F., Unger A., Sommerfeld L., Jansen J.M., Wagner U., Graumann J., Müller R., Reinartz S. (2020). Tumor-associated macrophages promote ovarian cancer cell migration by secreting transforming growth factor beta induced (TGFBI) and tenascin C. Cell Death Dis..

[65] Yao Z., Zhang J., Zhang B., Liang G., Chen X., Yao F., Xu X., Wu H., He Q., Ding L. (2018). Imatinib prevents lung cancer metastasis by inhibiting M2-like polarization of macrophages. Pharmacol. Res..

[66] Yang S., Fang Y., Ma Y., Wang F., Wang Y., Jia J., Yang Y., Sun W., Zhou Q., Li Z. (2025). Angiogenesis and targeted therapy in the tumour microenvironment: From basic to clinical practice. Clin. Transl. Med..

[67] Garlisi B., Lauks S., Aitken C., Ogilvie L.M., Lockington C., Petrik D., Eichhorn J.S., Petrik J. (2024). The Complex Tumor Microenvironment in Ovarian Cancer: Therapeutic Challenges and Opportunities. Curr. Oncol..

[68] Tariq M., Zhang J., Liang G., Ding L., He Q., Yang B. (2017). Macrophage Polarization: Anti-Cancer Strategies to Target Tumor-Associated Macrophage in Breast Cancer. J. Cell. Biochem..

[69] Bai B., Xie S., Wang Y., Wu F., Chen Y., Bian J., Gao X. (2024). Development of anti-cancer drugs for tumor-associated macrophages: A comprehensive review and mechanistic insights. Front. Mol. Biosci..

[70] Wang S., Wang J., Chen Z., Luo J., Guo W., Sun L., Lin L. (2024). Targeting M2-like tumor-associated macrophages is a potential therapeutic approach to overcome antitumor drug resistance. npj Precis. Oncol..

[71] Zhu J., Cai C., Li J., Xiao J., Duan X. (2022). CD47-SIRPα axis in cancer therapy: Precise delivery of CD47-targeted therapeutics and design of anti-phagocytic drug delivery systems. Med. Drug Discov..

[72] Huo X., Tian T., Zhang X., Zhou N. (2025). Comparative effectiveness and safety of treatment regimens for recurrent advanced ovarian cancer: A systematic review and network meta-analysis. World, J. Surg. Oncol..

[73] Shang Q., Zhang P., Lei X., Du L., Qu B. (2025). Insights into CSF-1/CSF-1R signaling: The role of macrophage in radiotherapy. Front. Immunol..

[74] Hume D.A., MacDonald K.P.A. (2012). Therapeutic applications of macrophage colony-stimulating factor-1 (CSF-1) and antagonists of CSF-1 receptor (CSF-1R) signaling. Blood.

[75] Anfray C., Ummarino A., Andón F.T., Allavena P. (2019). Current Strategies to Target Tumor-Associated-Macrophages to Improve Anti-Tumor Immune Responses. Cells.

[76] Yang Y.-I., Wang Y.-Y., Ahn J.-H., Kim B.-H., Choi J.-H. (2022). CCL2 overexpression is associated with paclitaxel resistance in ovarian cancer cells via autocrine signaling and macrophage recruitment. Biomed. Pharmacother..

[77] Su P., Li O., Ke K., Jiang Z., Wu J., Wang Y., Mou Y., Jin W. (2024). Targeting tumor-associated macrophages: Critical players in tumor progression and therapeutic strategies (Review). Int. J. Oncol..

[78] Meric-Bernstam F., Sweis R.F., Kasper S., Hamid O., Bhatia S., Dummer R., Stradella A., Long G.V., Spreafico A., Shimizu T. (2022). Combination of the STING Agonist MIW815 (ADU-S100) and PD-1 Inhibitor Spartalizumab in Advanced/Metastatic Solid Tumors or Lymphomas: An Open-Label, Multicenter, Phase Ib Study. Clin. Cancer Res..

[79] Graziani G., Tentori L., Navarra P. (2012). Ipilimumab: A novel immunostimulatory monoclonal antibody for the treatment of cancer. Pharmacol. Res..

[80] Wolchok J.D., Kluger H., Callahan M.K., Postow M.A., Rizvi N.A., Lesokhin A.M., Segal N.H., Ariyan C.E., Gordon R.-A., Reed K. (2013). Nivolumab plus Ipilimumab in Advanced Melanoma. N. Engl. J. Med..

[81] Lipson E.J., Drake C.G. (2011). Ipilimumab: An Anti-CTLA-4 Antibody for Metastatic Melanoma. Clin. Cancer Res..

[82] Robert C., Schachter J., Long G.V., Arance A., Grob J.J., Mortier L., Daud A., Carlino M.S., McNeil C., Lotem M. (2015). Pembrolizumab versus Ipilimumab in Advanced Melanoma. N. Engl. J. Med..

[83] Pu Y., Ji Q. (2022). Tumor-Associated Macrophages Regulate PD-1/PD-L1 Immunosuppression. Front. Immunol..

[84] Li S., Jiang B., Zhou H., Yang S., Yang L., Hong Y. (2025). Development of a prognostic immune cell-based model for ovarian cancer using multiplex immunofluorescence. J. Transl. Med..

[85] Rao R., Han R., Ogurek S., Xue C., Wu L.M., Zhang L., Zhang L., Hu J., Phoenix T.N., Waggoner S.N. (2021). Glioblastoma genetic drivers dictate the function of tumor-associated macrophages/microglia and responses to CSF1R inhibition. Neuro-Oncology.

[86] Wang W., Li T., Cheng Y., Li F., Qi S., Mao M., Wu J., Liu Q., Zhang X., Li X. (2024). Identification of hypoxic macrophages in glioblastoma with therapeutic potential for vasculature normalization. Cancer Cell.

[87] Pennisi G., Valeri F., Burattini B., Bruzzaniti P., Sturiale C.L., Talacchi A., Papacci F., Olivi A., Della Pepa G.M. (2025). Targeting Macrophages in Glioblastoma: Current Therapies and Future Directions. Cancers.

[88] Farhangnia P., Khorramdelazad H., Nickho H., Delbandi A.-A. (2024). Current and future immunotherapeutic approaches in pancreatic cancer treatment. J. Hematol. Oncol..

[89] Pan D., Li X., Qiao X., Wang Q. (2025). Immunosuppressive tumor microenvironment in pancreatic cancer: Mechanisms and therapeutic targets. Front. Immunol..

[90] Minaei E., Ranson M., Aghmesheh M., Sluyter R., Vine K.L. (2024). Enhancing Pancreatic Cancer Immunotherapy: Leveraging Localized Delivery Strategies through the Use of Implantable Devices and Scaffolds. J. Control. Release.

[91] Yu Z., Zou J., Xu F. (2024). Tumor-associated macrophages affect the treatment of lung cancer. Heliyon.

[92] Rannikko J.H., Hollmén M. (2024). Clinical landscape of macrophage-reprogramming cancer immunotherapies. Br. J. Cancer.

[93] Ding J., Guo C., Hu P., Chen J., Liu Q., Wu X., Cao Y., Wu J. (2016). CSF1 is involved in breast cancer progression through inducing monocyte differentiation and homing. Int. J. Oncol..

[94] Melaiu O., Vanni G., Portarena I., Pistolese C.A., Anemona L., Pomella S., Bei R., Buonomo O.C., Roselli M., Mauriello A. (2023). The Combination of Immune Checkpoint Blockade with Tumor Vessel Normalization as a Promising Therapeutic Strategy for Breast Cancer: An Overview of Preclinical and Clinical Studies. Int. J. Mol. Sci..

[95] Schweer D., McAtee A., Neupane K., Richards C., Ueland F., Kolesar J. (2022). Tumor-Associated Macrophages and Ovarian Cancer: Implications for Therapy. Cancers.

[96] McDermott M.S., O’BRien N.A., Hoffstrom B., Gong K., Lu M., Zhang J., Luo T., Liang M., Jia W., Hong J.J. (2023). Preclinical Efficacy of the Antibody–Drug Conjugate CLDN6–23-ADC for the Treatment of CLDN6-Positive Solid Tumors. Clin. Cancer Res..

[97] Wang Y., Ma C., Li X., Yang F., Wang N., Ji G., Liu Q., Zhu H., Xu S., Li H. (2025). Unraveling the role of M2 TAMs in ovarian cancer dynamics: A systematic review. J. Transl. Med..

[98] Liu M., Liu L., Song Y., Li W., Xu L. (2022). Targeting macrophages: A novel treatment strategy in solid tumors. J. Transl. Med..

[99] Yang M., Li Z., Ren M., Li S., Zhang L., Zhang X., Liu F. (2018). Stromal Infiltration of Tumor-Associated Macrophages Conferring Poor Prognosis of Patients with Basal-Like Breast Carcinoma. J. Cancer.

[100] Karwicka K., Wawer J., Czabak O., Kocki J., Hus M. (2020). Innowacyjna terapia CAR-T w leczeniu nowotworów hematologicznych—Wybrane aspekty genetyczne i immunologiczne. Hematologia.

[101] Jackson H.J., Rafiq S., Brentjens R.J. (2016). Driving CAR T-cells forward. Nat. Rev. Clin. Oncol..

[102] Chmielewski M., Abken H. (2015). TRUCKs: The fourth generation of CARs. Expert Opin. Biol. Ther..

[103] Koneru M., Purdon T.J., Spriggs D., Koneru S., Brentjens R.J. (2015). IL-12 secreting tumor-targeted chimeric antigen receptor T cells eradicate ovarian tumors in vivo. OncoImmunology.

[104] Andreou T., Neophytou C., Mpekris F., Stylianopoulos T. (2025). Expanding Immunotherapy Beyond CAR T Cells: Engineering Diverse Immune Cells to Target Solid Tumors. Cancers.

[105] Li X., Wang X., Wang H., Zuo D., Xu J., Feng Y., Xue D., Zhang L., Lin L., Zhang J. (2024). A clinical study of autologous chimeric antigen receptor macrophage targeting mesothelin shows safety in ovarian cancer therapy. J. Hematol. Oncol..

[106] Nonaka K., Saio M., Suwa T., Frey A.B., Umemura N., Imai H., Ouyang G.-F., Osada S., Balazs M., Adany R. (2008). Skewing the Th cell phenotype toward Th1 alters the maturation of tumor-infiltrating mononuclear phagocytes. J. Leukoc. Biol..

[107] Henze A.T., Mazzone M. (2016). The impact of hypoxia on tumor-associated macrophages. J Clin Invest..

[108] Klichinsky M., Ruella M., Shestova O., Lu X.M., Best A., Zeeman M., Schmierer M., Gabrusiewicz K., Anderson N.R., Petty N.E. (2020). Human chimeric antigen receptor macrophages for cancer immunotherapy. Nat. Biotechnol..

[109] Reiss K.A., Angelos M.G., Dees E.C., Yuan Y., Ueno N.T., Pohlmann P.R., Johnson M.L., Chao J., Shestova O., Serody J.S. (2025). CAR-macrophage therapy for HER2-overexpressing advanced solid tumors: A phase 1 trial. Nat. Med..

[110] Sloas C., Gill S., Klichinsky M. (2021). Engineered CAR-Macrophages as Adoptive Immunotherapies for Solid Tumors. Front. Immunol..

[111] Wang S., Yang Y., Ma P., Zha Y., Zhang J., Lei A., Li N. (2022). CAR-macrophage: An extensive immune enhancer to fight cancer. EBioMedicine.

[112] June C.H., O’Connor R.S., Kawalekar O.U., Ghassemi S., Milone M.C. (2018). CAR T cell immunotherapy for human cancer. Science.

[113] Kelly P., Davison R., Bliss E., McGee J. (1988). Macrophages in human breast disease: A quantitative immunohistochemical study. Br. J. Cancer.

[114] Huang Z., Sun X., Liu X., Shen Y., Wang K. (2017). Macrophages as an active tumour-targeting carrier of SN38-nanoparticles for cancer therapy. J. Drug Target..

[115] Muthana M., Giannoudis A., Scott S.D., Fang H.-Y., Coffelt S.B., Morrow F.J., Murdoch C., Burton J., Cross N., Burke B. (2011). Use of Macrophages to Target Therapeutic Adenovirus to Human Prostate Tumors. Cancer Res..

[116] Muthana M., Kennerley A.J., Hughes R., Fagnano E., Richardson J., Paul M., Murdoch C., Wright F., Payne C., Lythgoe M.F. (2015). Directing cell therapy to anatomic target sites in vivo with magnetic resonance targeting. Nat. Commun..

[117] Chernajovsky Y., Layward L., Lemoine N. (2006). Fighting cancer with oncolytic viruses. BMJ.

[118] Choi J., Kim H.-Y., Ju E.J., Jung J., Park J., Chung H.-K., Lee J.S., Lee J.S., Park H.J., Song S.Y. (2012). Use of macrophages to deliver therapeutic and imaging contrast agents to tumors. Biomaterials.

[119] Taciak B., Bialasek M., Kubiak M., Marszalek I., Gorczak M., Osadchuk O., Kurpiel D., Strzemecki D., Barwik K., Skorzynski M. (2025). Harnessing macrophage-drug conjugates for allogeneic cell-based therapy of solid tumors via the TRAIN mechanism. Nat. Commun..

[120] Magdalena K., Irene B., Paola B., Tomasz R., Alberto B. (2022). Cellular Targeted Active Ingredient Delivery System. U.S. Patent.

[121] Sun M., Bialasek M., Mayoux M., Lin M.-S., Buck A., Marszałek I., Taciak B., Bühler M., Górczak M., Kucharzewska P. (2025). Adoptive cell therapy with macrophage-drug conjugates facilitates cytotoxic drug transfer and immune activation in glioblastoma models. Sci. Transl. Med..

[122] Meyron-Holtz E.G., Fibach E., Gelvan D., Konijn A.M. (1994). Binding and uptake of exogenous isoferritins by cultured human erythroid precursor cells. Br. J. Haematol..

[123] Morva A., Arroyo A.B., Andreeva L., Tapia-Abellán A., Luengo-Gil G. (2025). Unleashing the power of CAR-M therapy in solid tumors: A comprehensive review. Front. Immunol..

